# Daily, self-test rapid antigen test to assess SARS-CoV-2 viability in de-isolation of patients with COVID-19

**DOI:** 10.3389/fmed.2022.922431

**Published:** 2022-10-19

**Authors:** Seongman Bae, Heedo Park, Ji Yeun Kim, Sunghee Park, So Yun Lim, Joon-Yong Bae, Jeonghun Kim, Jiwon Jung, Min Jae Kim, Yong Pil Chong, Sang-Oh Lee, Sang-Ho Choi, Yang Soo Kim, Man-Seong Park, Sung-Han Kim

**Affiliations:** ^1^Department of Infectious Diseases, Asan Medical Center, University of Ulsan College of Medicine, Seoul, South Korea; ^2^BK21 Graduate Program, Department of Biomedical Sciences, Korea University College of Medicine, Seoul, South Korea; ^3^Department of Microbiology, Biosafety Center, College of Medicine, Institute for Viral Diseases, Korea University, Seoul, South Korea

**Keywords:** COVID-19, rapid antigen test, isolation, de-isolation, cell culture

## Abstract

**Background:**

Isolation of COVID-19 patients is a crucial infection control measure to prevent further SARS-CoV-2 transmission, but determining an appropriate timing to end the COVID-19 isolation is a challenging. We evaluated the performance of the self-test rapid antigen test (RAT) as a potential proxy to terminate the isolation of COVID-19 patients.

**Materials and methods:**

Symptomatic COVID-19 patients were enrolled who were admitted to a regional community treatment center (CTC) in Seoul (South Korea). Self-test RAT and the collection of saliva samples were performed by the patients, on a daily basis, until patient discharge. Cell culture and subgenomic RNA detection were performed on saliva samples.

**Results:**

A total of 138 pairs of saliva samples and corresponding RAT results were collected from 34 COVID-19 patients. Positivity of RAT and cell culture was 27% (37/138) and 12% (16/138), respectively. Of the 16 culture-positive saliva samples, seven (43.8%) corresponding RAT results were positive. Using cell culture as the reference standard, the overall percent agreement, percent positive agreement, and percent negative agreement of RAT were 71% (95% CI, 63–78), 26% (95% CI, 12–42), and 82% (95% CI, 76–87), respectively. The sensitivity, specificity, positive predictive value, and negative predictive value of the RAT for predicting culture results were 44% (95% CI, 20–70), 75% (95% CI, 66–82), 18% (95% CI, 8–34), and 91% (95% CI, 84–96), respectively.

**Conclusion:**

About half of the patients who were SARS-CoV-2 positive based upon cell culture results gave negative RAT results. However, the remaining positive culture cases were detected by RAT, and RAT showed relatively high negative predictive value for viable viral shedding.

## Introduction

Despite the introduction of COVID-19 vaccines, a large number of new patients worldwide are still being infected with SARS-CoV-2 due to the emergence of virus variants or vaccine shortages (1). Along with the rapid distribution of vaccines, proactive testing, contact tracing, and isolation of confirmed COVID-19 patients are still key elements of infection control measures. Many countries, including South Korea, are adopting symptom-based isolation strategies that require isolating COVID-19 patients with mild to moderate symptoms, but without immunocompromising conditions, for at least 10 days after symptom onset until clinical improvement is achieved (2). However, uniform application of the symptom-based isolation strategy entails social costs since some individuals lose their infectivity before 10 days (3). Furthermore, due to the recent emergence of the Omicron variant, the isolation period has been further curtailed from 10 to 5 days in patients with asymptomatic or mild COVID-19 (4). Since there are concerns about the residual infectivity associated with early de-isolation, a rapid antigen test (RAT)-based de-isolation strategy has been endorsed by CDC and European CDC guidelines (4, 5). Despite lower sensitivity of RAT for diagnosing acute SARS-CoV-2 infection compared to nucleic acid amplification testing (NAAT) such as RT-PCR, positive RAT results correlated well with high viral load samples (6). Therefore, positive RAT results were also expected to correlate with positive viral culture, which have been considered a proxy for infectivity (7). However, there are limited studies comparing the results of serially performed RATs during infection with tests for infectivity, such as virus culture (8, 9). In this study, the results of serially performed, self-test RAT were compared with those of virus culture, genomic RNA, and subgenomic RNA tests on saliva samples collected from COVID-19 patients in South Korea.

## Materials and methods

### Study population and setting

In South Korea, in 2021, asymptomatic or mild symptomatic patients, who were newly diagnosed with COVID-19, were admitted to a community treatment center (CTC) to prevent further spread of SARS-CoV-2, and to monitor the clinical course of COVID-19 (10, 11). Patients who were at high risk of progressing to severe COVID-19, such as the elderly (over 70 years old) and immunocompromised patients, were admitted to a dedicated hospital facility rather than a CTC. According to the government guidelines for COVID-19 patients, all new SARS-CoV-2 patients should be isolated in a CTC or hospital facilities for at least 10 days if symptoms have resolved. During admission to a CTC, patients were asked to self-check their vital signs (body temperature, oxygen saturation, blood pressure, etc.) using portable medical devices and report these data, along with any COVID-19 related symptoms, to the medical staff twice a day. Patients who reported respiratory distress, intractable fever, or desaturation were transported to the hospital as they were considered at risk of progression to severe COVID-19.

This observational study enrolled patients infected with SARS-CoV-2 who were admitted to the University of Seoul CTC (Seoul, South Korea) from June 21, 2021 to August 21, 2021. COVID-19 was confirmed by RT-PCR in all enrolled patients. All patients participated voluntarily and provided written informed consent prior to enrollment. Participants were asked to perform a self-test RAT and collect saliva on a daily basis. The presence of SARS-CoV-2 in saliva samples was detected using RT-PCR (based on both genomic and subgenomic RNA sequences of SARS-CoV-2) and cell culture. The results of tests performed on saliva samples were then compared with the RAT results. The study protocol was approved by the institutional review committee of Asan Medical Center (IRB no. 2020-0336), which oversees the operation of the CTC.

### Rapid antigen testing

In this study, the Humasis COVID-Ag Home Test kit (Humasis Co., South Korea) was used for serial self-RAT testing. This RAT is a lateral flow immunochromatographic assay for the qualitative detection of nucleocapsid protein and receptor binding domain (RBD) antigens of SARS-CoV-2. This assay was approved as a screening test for COVID-19 by the Ministry of Food and Drug Safety in South Korea. Tests were performed by patients, according to the manufacturer’s protocol; briefly, self-collected nasal swabs from both nares were placed in extraction solution, swirled ten times, and squeezed against the collection tube wall. Extracted sample was applied to a cassette, and an appropriate time was allowed for a monoclonal anti-SARS-CoV-2 antibody reaction. Test results were interpreted after 15 min. There are two lines on the cassette: a colored control line (C) should always appear after adding an appropriate sample volume. A positive result was defined as a colored band at the *T*-test mark on the cassette, regardless of whether it was weak or clear. Negative results were indicated by the absence of a band at the T mark. If the control reaction failed, the test was considered invalid and was repeated. The results were read by two independent observers.

### Collection of daily saliva samples

Self-collected saliva samples were obtained from patients from the day of study enrollment until the day of discharge. Each day, patients collected a 2 mL volume of saliva into an airtight container provided at admission; no preservation or transport medium was used. Patients were asked to avoid food, water, and teeth brushing for at least 30 min prior to sample collection. Saliva samples were collected within 1 h by medical staff and transported to a designated laboratory where they were aliquoted and stored at −80°C until use.

### Measurement of viral load by real-time RT-PCR assay

The collected saliva samples were inactivated at 65°C for 30 min in a negative pressure laboratory. Viral RNA was extracted from saliva samples using a QIAamp viral RNA Mini kit (Qiagen Inc., Hilden, Germany). To determine the SARS-CoV-2 viral RNA copy number, multiplex real-time RT-PCR assays targeting the S- and N-genes were developed. Multiplex RT-PCR assay mix (20 μL) contained 4 μL of 5 × master mix (LightCycler Multiplex RNA Virus Master, Roche, Basel, Switzerland), 0.1 μL of 200 × enzyme mix, 500 nM of each S and N gene primer, 200 nM of each S and N gene probe, 250 nM of internal control primers, 100 nM of internal control probes, and 5 μL of extracted RNA or *in vitro*-synthesized control RNA. PCR amplification was performed with a LightCycler 96 system (Roche) in the following conditions: reverse transcription at 50°C for 10 min, initial denaturation at 95°C for 5 min, 45 cycles of two-step amplification, denaturation at 95°C for 10 s, and final extension at 60°C for 30 s. To generate calibration curves, serial dilutions from 10^7^ to five copies/μL of synthetic control RNA were assayed in six independent sets of reactions (Supplementary Figure 1). The detection limit of this assay was five copies/reaction (2.6 log copies/ml of specimen), and viral copy numbers were determined by plotting CT values against log copies/reaction. The primer and probe sequences are provided in (Supplementary Table 1).

### Detection of N and S gene subgenomic RNAs

SARS-CoV-2 N and S gene subgenomic RNAs were detected using RocketScript RT-PCR Premix (Bioneer Co., Daejeon, South Korea). The shared forward primer was designed in the 5′ leader sequence, and reverse primers were located in the gene sequences encoding the N- and S-proteins (Supplementary Table 2). In brief, RT-PCR reactions were performed as follows: reverse transcription at 50°C for 30 min, initial denaturation at 95°C for 5 min, 40 cycles of three-step amplification, denaturation at 95°Cfor 30 s, annealing at 55°Cfor 30 s, extension at 72°C for 1 min, and final extension at 72°C for 5 min. Amplification products were eluted with a QIAquick Gel Extraction kit (Qiagen), and sequencing was carried out by Macrogen Inc. (Seoul, Republic of Korea). Sequences that included the leader sequence and that were ≥97% consistent with the SARS-CoV-2 genome, by BLAST, were confirmed as subgenomic RNAs.

### Cell culture

Culture-based isolation of SARS-CoV-2 from saliva was performed by a plaque assay in a Biosafety Level 3 laboratory at Korea University College of Medicine, Seoul, South Korea. Vero cells (9 × 10^5^ cells/well) were seeded into 6-well plates and allowed to incubate for 24 h. Saliva specimens were serially 10-fold diluted using PBS. Aliquots (200 μl) of each diluted sample were inoculated onto the Vero cells and incubated for 1 h (37°C, 5% [v/v] CO_2_) with rocking every 15 min and overlaid with 2 mL of Dulbecco’s Modified Eagle Medium/Nutrient Mixture F12 (DMEM/F-12) medium containing 0.6% (w/v) oxoid agar. Viral plaque formation was visualized by crystal violet staining after 72 h of incubation at 37°C in a 5% (v/v) CO_2_ incubator.

### Statistical analyses

Categorical variables were described as number with percentage, and continuous variables were described as mean with standard deviation or median with interquartile range or range, as appropriate. Percent agreement between the results of the self-test RAT and virus culture was calculated as numbers of concordant pairs divided by total number of paired observations. The percent positive agreement was calculated by dividing the number of observations that were positive for both tests by the average of the number of positive observations in each test. The percent negative agreement was calculated by dividing the number of observations that were negative for both tests by the average of the number of negative observations in each test. The sensitivity, specificity, positive predictive value, and negative predictive value of results of COVID-19 self-test RAT were estimated with positive results of virus culture or subgenomic RNA from saliva samples as reference standards. Data were analyzed using R version 4.0.4 (R Project for Statistical Computing, Vienna, Austria).

## Results

The baseline characteristics of the 34 patients with symptomatic COVID-19 who enrolled in this study are summarized in Table 1. The mean age was 31.8 years, and 61.8% were male. Most patients (85% [29/34]) were admitted to the CTC within a day or two after diagnosis. The median time from symptom onset and admission to the day of first RAT testing were 5 (interquartile range [IQR], 4–6) and 3 days (IQR, 3–3), respectively. No abnormal infiltration was observed except for one patient on chest imaging performed on the day of admission. All patients were clinically recovered at discharge, but one (2.9%) was transferred to hospital due to intractable fever. The median time between admission and discharge was 10 days (range, 5–14). The median value of viral load at diagnosis was 18.2, and the majority of cases (73.5%) were Delta variants. During the study period, a total of 151 RAT results and 138 saliva samples were collected, resulting in 138 paired RAT results and saliva samples. The median time to negative RAT result was 4 days (interquartile range [IQR], 3–6) from admission and 7.5 days (IQR, 6–8) from symptom onset (Supplementary Figure 2).

**TABLE 1:** Baseline characteristics of patients.

	Patients (*N* = 34)
Age, mean ± SD, year	31.8 ± 9.0
Male, no (%)	21 (61.8)
**Comorbidity, no (%)**	
Diabetes	1 (2.9)
Hypertension	1 (2.9)
Asthma	1 (2.9)
Depression	3 (8.8)
**Patient classification by symptom**	
Symptomatic	32 (94.1)
Presymptomatic	2 (5.9)
Asymptomatic	0 (0)
**Symptoms at admission**	
Fever	26 (76.5)
Chill	9 (26.5)
Cough	17 (50.0)
Sputum	5 (14.7)
Sore throat	17 (50.0)
Dyspnea	1 (2.9)
Rhinorrhea	4 (11.8)
Nasal stuffiness	4 (11.8)
Myalgia	17 (50.0)
Headache	9 (26.5)
Loss of taste	1 (2.9)
Loss of smell	4 (11.8)
Diarrhea	1 (2.9)
Days from symptom onset to admission*, median (range)	2 (0–8)
Days from COVID-19 diagnosis to admission*, median (range)	1 (0–2)
Days from symptom onset to first RAT test, median (IQR)	5 (4–6)
Days from admission to first RAT test, median (IQR)	3 (3–3)
Median days from admission to discharge, no. (range)	10 (5–14)
Mean viral load at diagnosis, Ct value (E gene)^†^	18.2
Infiltrations on chest x-ray at admission, no (%)	1 (2.9)
**Delta variant (%)**	
Yes	25 (73.5)
No	9 (26.5)

IQR, interquartile range. *Admission indicates admission to a community treatment center for isolation purposes. ^†^Initial viral load at the time of diagnosis (one missing).

### Results and predictive performance of rapid antigen test compared with viral culture and subgenomic RNA

**FIGURE 1:**
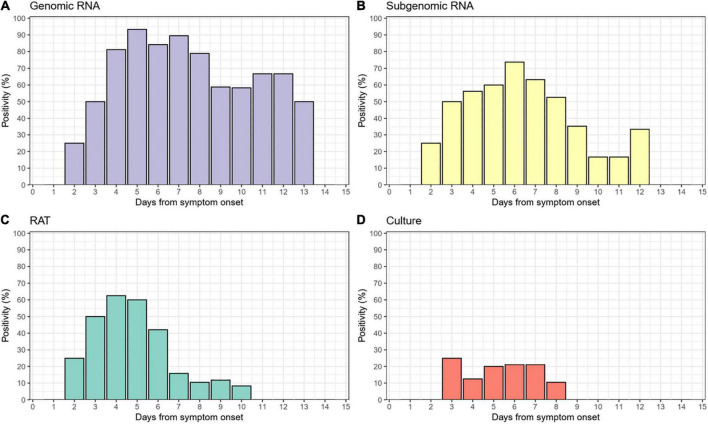
Daily positivity of **(A)** genomic RNA, **(B)** subgenomic RNA, **(C)** rapid antigen test (RAT), and **(D)** cell culture.

**FIGURE 2:**
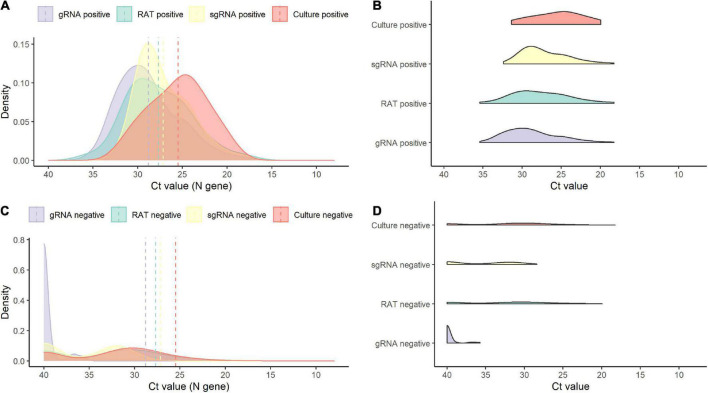
Density plots of positive and negative results for each test. **(A)** Density plot for positive results by test. **(B)** Raincloud plot for positive results by each test. **(C)** Density plot for negative results by test. **(D)** Raincloud plot for negative results by each test. sgRNA, subgenomic RNA; gRNA, genomic RNA.

Of the 138 paired RAT results and saliva samples tested, 27.5% (38/138) of RAT, 11.6% (16/138) of cell culture, and 48.6% (67/138) of subgenomic RNA tests were positive for SARS-CoV-2 (Table 2). Of the 16 culture-positive saliva samples, seven (43.8%) corresponding RAT results were also positive. The daily, positive rates using RAT, genomic RNA, subgenomic RNA, and cell culture gradually decreased with time from 5 days after symptom onset (Figure 1). The overall percent agreement, percent positive agreement, and percent negative agreement between RAT and viral culture were 71% (95% CI, 63–78), 26% (95% CI, 12–42), and 82% (95% CI, 76–87), respectively. Of the 67 subgenomic RNA-positive samples, 30 (44.8%) were also positive in paired RAT results. The overall percent agreement, percent positive agreement, and percent negative agreement between RAT and subgenomic RNA test were 67% (95% CI, 60–75), 57% (95% CI, 45–68), and 74% (95% CI, 66–80), respectively. The mean Ct values for positive samples were highest in viral culture, followed by subgenomic RNA, RAT, and genomic RNA tests (Figure 2). The viral load (median log_10_ copies/mL, [interquartile range]) was significantly different according to positivity for RAT (4.8 [IQR, 4.1–5.7] vs. 3.6 [1.3–4.5]), culture (5.8 [4.9–6.3] vs. 3.9 [1.3–4.6]), and subgenomic RNA (5.0 [4.5–5.7] vs. 1.3 [1.3–3.6]; all *P* < 0.001) as shown in Supplementary Figure 3.

**TABLE 2:** Performance of self-test rapid antigen tests compared with viral culture, subgenomic RNA, and genomic RNA.

	No. of pairs	Sensitivity (95% CI)	Specificity (95% CI)	PPV (95% CI)	NPV (95% CI)
**RAT vs. viral culture**					
Overall	138	44% (20–70)	75% (66–82)	18% (8–34)	91% (84–96)
≤5 days	40	83% (36–100)	50% (32–68)	23% (8–45)	94% (73–100)
>5 days	98	20% (3–56)	84% (75–91)	12% (2–38)	90% (82–96)
**RAT vs. sgRNA**					
Overall	138	45% (33–57)	89% (79–95)	79% (63–90)	63% (53–72)
≤5 days	40	81% (58–95)	74% (49–91)	77% (55–92)	78% (52–94)
>5 days	98	28% (16–43)	94% (84–99)	81% (54–96)	60% (48–70)
**RAT vs. gRNA**					
Overall	138	37% (28–47)	100% (90–100)	100% (91–100)	36% (27–46)
≤5 days	40	73% (54–88)	100% (69–100)	100% (85–100)	56% (31–78)
>5 days	98	22% (13–34)	100% (87–100)	100% (79–100)	32% (22–43)

CI, confidence interval; PPV, positive predictive value; NPV, negative predictive value; sgRNA, subgenomic RNA; gRNA, genomic RNA.

The performance of RAT for predicting positive results in viral culture, subgenomic RNA, and genomic RNA is summarized in Table 2. The sensitivity, specificity, positive predictive value, and negative predictive value of the RAT for predicting the results of viral culture were 44% (95% CI, 20–70), 75% (95% CI, 66–82), 18% (95% CI, 8–34), and 91% (95% CI, 84–96), respectively (Table 2). The sensitivity, specificity, positive predictive value, and negative predictive value of the RAT for predicting positive subgenomic RNA detection was 45% (95% CI, 33–57), 89% (95% CI, 79–95), 79% (95% CI, 63–90), and 63% (95% CI, 53–72), respectively. The sensitivity of RAT to viral culture increased to 83% (95% CI, 36–100) when applied to samples collected up to 5 days after symptom onset, and decreased to 20% (3–56) when applied to samples collected 5 days after symptom onset. The sensitivity of RAT to subgenomic RNA and genomic RNA was also higher when applied to samples collected up to 5 days after symptom onset than when applied to samples collected 5 days after symptom onset. The performances of genomic RNA PCR and subgenomic RNA PCR for predicting viral culture results are summarized in Supplementary Table 3.

### Results of rapid antigen test, culture, and subgenomic RNA according to the timeline

**FIGURE 3:**
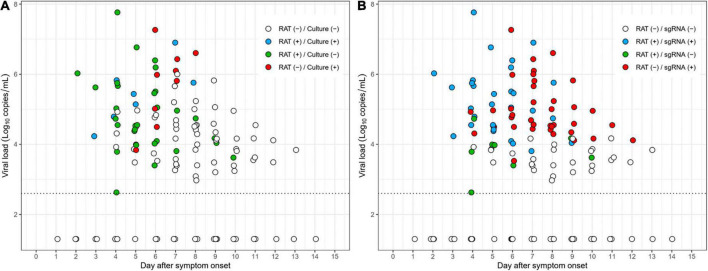
Scatter plots depicting RAT results over the study period. **(A)** Results of RAT compared to cell culture. **(B)** Results of RAT compared to subgenomic RNA.

As shown in Figure 3A, most culture-positive cases (83% [5/6]) were also positive with RAT (blue dots) up to 5 days after symptom onset, whereas most culture-positive cases (80% [8/10]) after 5 days were negative with RAT (red dots). Similarly, most subgenomic RNA-positive cases (81% [17/21]), up to 5 days after symptom onset, were also positive for RAT (blue dots), whereas the majority of the subgenomic RNA-positive cases (72% [33/46]) were negative with RAT (red dots) after 5 days (Figure 3B). Detailed scatter plots according to positivity of the reference tests are shown in Supplementary Figure 4.

**FIGURE 4:**
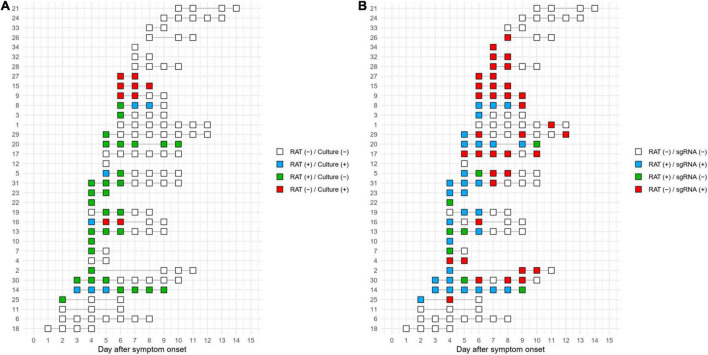
Timeline of the test results for RAT compared to cell culture **(A)** or subgenomic RNA **(B)**. The numbers on the *y*-axis represent the individual patient numbers.

Timelines of the test results at the individual patient level are shown in Figure 4. Of 7 patients with positive culture results, 4 of whom also had positive RAT results. In detail, four concordant pairs of positive culture with positive RAT (blue rectangle) and four discordant pairs of positive culture with negative RAT (red rectangle) were found (Figure 4A). A concordant pair was observed in one patient (Patient 16), but later discordant pairs were observed. On the basis of RAT-determined termination of isolation, the termination of three out of seven patients with culture-positive samples would have been delayed due to RAT results that predicted positive cultures, whereas RAT could have predict positive cultures in the remaining four patients. Using subgenomic RNA detection as the reference, RAT predicted positive subgenomic RNA in 15 of 25 subgenomic RNA-positive patients but failed to predict positive subgenomic RNA in subsequent samples from seven duplicates of these patients (Figure 4B). In the remaining ten subgenomic RNA-positive patients, RAT did not predict subgenomic RNA positivity. Results for RAT and cell culture for each patient are shown in Supplementary Figure 5.

## Discussion

In this longitudinal study in symptomatic COVID-19 patients, overall agreement between results of RAT and culture was fair at about 70%, but RAT detected culture-positive cases in less than half of the patients. These results, comparing serially self-performed RAT to cell culture from saliva samples, indicates suboptimal sensitivity of RAT for detecting viable viral shedding after diagnosis or symptom onset in COVID-19 patients. Nonetheless, RAT still detected about half of COVID-19 patients with viable viral shedding. Consequently, RAT results could be used for the risk stratification on work restriction of healthcare workers (HCWs), when there is high pressure on healthcare systems, because RAT would detect half of the HCWs with viable viral shedding and who have theoretical risk of post-isolation transmission while no test cannot detect them. This approach may be particularly useful for HCWs who care for immunocompromised patients. Alternatively, the relatively high negative predictive value of RAT may help to allay concerns about the transmission risk of individuals within contingency or crisis settings.

Conventional PCR tests (real-time RT-PCR assays) have the highest sensitivity in diagnosing SARS-CoV-2 infection, but also have several shortcomings including cost, long turnaround time, and prolonged test positivity without viable virus (12–16). PCR-based isolation strategies that maintain isolation until PCR results become negative have been increasingly limited in use due to their unnecessarily long isolation requirements. Symptom-based isolation strategies have been adopted based on previous studies that reported the detection of viable virus did not exceed 10 days, and that no case of secondary attack was shown among close contacts exposed to an index case 5 days after symptom onset (17, 18). However, symptom-based isolation strategies for a pre-specified period are not applicable in populations with prolonged viral shedding, such as severe COVID-19 patients or immunocompromised hosts, and also unnecessarily constrains the social activity of asymptomatic or mild COVID-19 patients whose release of viable virus has ended earlier than the time of their de-isolation. Furthermore, due to the recent emergence of the Omicron variant, the recommended isolation period has been further curtailed from 10 to 5 days in patients with asymptomatic or mild COVID-19 (4). Therefore, there has been a growing public need for tools that can be used as surrogate markers to identify individuals having transmissibility.

Rapid antigen test are intended for use at the point-of-care to detect the presence of viral protein of SARS-CoV-2 and are quick and easy to use, as well as relatively cost-affordable. Although the performance of RAT may vary by company, several studies reported its high sensitivity and specificity (6). It has been reported that the RAT positivity reflects high viral load and correlates well with culture positivity (19, 20). However, RAT showed low sensitivity, detecting only about half of the virus culture-positive samples in this study. The reason for the low sensitivity of RAT test can be demonstrated as follows. First, virus culture was used as a reference test for infectivity, but lack of sensitivity may lead to false-negative results (21). In this context, the detection of subgenomic RNA might more exactly reflect the replication-compatible viral shedding (Table 2). It is worth to note that our findings of the sensitivity (94%) and specificity (57%) for the subgenomic RNA detection compared with cell culture, respectively, are consistent with our previous study (sensitivity 100% and specificity 65%, respectively) (22). Second, the timing of the sample collection may affect the results. Given the viral kinetics of SARS-CoV-2 with a gradual decrease in viral load after the time of diagnosis (23), RAT generally performed well in samples containing high viral titers from symptomatic patients at an early stage (24, 25). Therefore, RAT performance may be lower on serial testing that included samples with low viral titer collected at later stages of infection. Third, difference between RAT with nasal swabs and other viral tests using saliva samples may contribute to the results, despite the high correlation between saliva and nasal swabs (26). Finally, sub-optimal sampling and misinterpreting results in in self-testing can affect the sensitivity of the RAT.

Despite this limited performance, RAT-positive samples showed significantly higher viral load than RAT-negative samples. In addition, the RAT-positive rate gradually decreased over time after symptom onset. At the time of this writing, Cosimi et al. reported that RAT has a high negative predictive value (100%), so a negative RAT result could provide reassurance in ending isolation (8). Our findings of high negative predictive value of a negative RAT are consistent with this study (8). In addition, CDC recently recommended continuation of wearing masks around others in public places until two consecutive negative RAT results (27). Taken together, RAT may detect replication-competent SARS-CoV-2 virus, and accuracy of this test can be improved by increasing the frequency or providing adequate guidance for procedure and interpretation (13, 28). Our data on the daily RAT results provide important insights into the contingency or crisis plan during the pandemic. More than half of mild COVID-19 patients revealed positive RAT results 5 days after the onset of symptoms. Consequently, when the strategy for 5 day isolation with a negative RAT result is adopted in a hospital, more than 50% of HCWs would be required to undertake a further isolation period. In addition, the low positive predictive value of RAT might warrant further balancing of work restriction. By contrast, the relatively high negative predictive value of RAT may allay concerns about the transmission risk posed by individuals in contingency or crisis setting because the prevalence of viable viral shedding is low, after symptom onset, in patients with mild COVID-19.

Cell culture has been considered the standard test for SARS-CoV-2 viability, but can only be performed in a biocontainment facility and is time and labor intensive (29, 30). Furthermore, culture is vulnerable to bacterial contamination. Detecting subgenomic RNA showed a higher specificity to predict culture positivity than that of genomic RNA, and was closely correlated with symptom duration, suggesting that it may reflect the presence a replication-competent virus (22). Since viral culture lacks sensitivity and may underestimate the level of viable virus, we compared RAT results with the subgenomic RNA detection data. These analyses revealed that the positive predictive value of RAT increased, but the negative predictive value of RAT decreased, largely due to the positive rate of subgenomic RNA detection being higher than that of viral culture. Given that subgenomic RNA detection is more sensitive for viable viral shedding than viral culture, and a highly sensitive test of viable viral shedding is needed in certain settings (e.g., immunocompromised patient wards), the greater positive predictive value of RAT may point to such tests being more beneficial in high-risk rather than low-risk settings.

It is worth noting that demonstrating the presence of viable virus by cell culture or replication-competent virus by subgenomic RNA detection does not necessarily correlate with transmissibility potential. The current CDC and ECDC recommendations are primarily based on epidemiological data showing that there is no significant risk of SARS-CoV-2 transmission from index patients, 3 or 5 days after symptom onset, to exposed contacts (18, 31). However, such epidemiologic data may be subject to recall and misclassification bias. Therefore, the study of viable viral shedding might provide important complementary data for understanding viral transmission dynamics. In this context, our data showing daily positive rates of viral cultures along with a series of self-test RAT results may be useful for the decision of symptom-based de-isolation or work derestriction with/without supplemental tests.

There are several limitations of this study. Firstly, it is an observational study with a limited sample size. In addition, there were some missing results from serially collected RAT results and saliva samples. Thus, further well-controlled studies with larger sample sizes are needed. Secondly, the fact that patients performed the RAT without guidance from medical professionals may account for the lower-than-expected predictive accuracy of RAT. Given the nature of the at-home test kit, user-dependent variability may be an inherent feature of studies utilizing at-home test kits. Thus, the safe, reliable and accurate termination of COVID-19 isolation based upon RAT results, may necessitate the execution of the RAT by healthcare professionals, although many countries have approved the RAT as “home use” only. Despite the imperfection of self-testing, at-home RAT will be needed continuously considering the importance of early diagnosis of SARS-CoV-2 infection and inequality in accessibility/cost/time according to region and economic status (32, 33). Thirdly, the correlation between RAT results and cell culture/subgenomic RNA results may differ for other commercial SARS-CoV-2 RAT kits. In two independent evaluation studies, the RAT from the same manufacturer (Humasis Co., South Korea), although not the at-home kit used in this study, showed similar sensitivities compared to RATs from other manufacturers, but with lower specificity, from 72.8 to 79.0% (34, 35). Such low specificity may raise concerns about an increased risk for false positives. It is unlikely that the insufficient specificity of the RAT was due to detection of spike antigens in addition to nucleocapsid, because targeting spike antigen could be more specific than nucleocapsid (36). Given the 100% specificity of the Humasis RAT in the current study and the very low false-positive rate (0.05%) of the RAT reported in a recent large study, the low specificity issue does not appear to significantly affect the results of this study (37). In addition, the recent study reported the manufacturing issues as one cause of the cluster of false-positive RAT results (37). Further studies are needed as false positives can be attributed to multiple factors such as batch issues, cross-contamination, pre-existing human antibodies, or highly viscous samples. Fourthly, this study was conducted during the Delta variant epidemic, so it is not known whether the results can be applied to analysis of the Omicron variant. A study carried out during the Omicron variant epidemic reported that the RAT used did detect viral protein of that variant (38). However, there are no data on whether RAT results significantly differ between the variants. Finally, only a few vaccinated patients were included in this study. However, vaccine status is unlikely to affect the results of RAT, even though it does affect viral load kinetics (39, 40).

In conclusion, about half of the patients in this study who shed viable virus after symptom onset returned negative RAT results. Therefore, a negative RAT result cannot be used as a guarantee test for non-infectivity. Nevertheless, the remaining patients with viable virus shedding were identified by positive RAT results, and RAT exhibited relatively high negative predictive value for viable viral shedding. Consequently, RAT may provide an additional layer of data to identify individuals with risk of infectivity in symptom-based de-isolation strategies.

## Data availability statement

The raw data supporting the conclusions of this article will be made available by the authors, without undue reservation.

## Ethics statement

The studies involving human participants were reviewed and approved by the Institutional Review Committee of Asan Medical Center. The patients/participants provided their written informed consent to participate in this study.

## Author contributions

SB, M-SP, and S-HK contributed to the conception and design of the study. SB, SP, and SL involved in participant recruitment and data curation. JYK, HP, J-YB, JK, and M-SP performed laboratory works. SB, SP, SL, and JYK performed formal analysis. SB, HP, M-SP, and S-HK wrote the first draft of the manuscript. MK, YC, SL, SC, and YK wrote sections of the manuscript. All authors contributed to the manuscript revision, read, and approved the submitted version.
