# Supplementary material for: Daily, self-test rapid antigen test to assess SARS-CoV-2 viability in de-isolation of patients with COVID-19
Source: Front Med (Lausanne). 2022 Oct 19;9:922431. doi: 10.3389/fmed.2022.922431 (PMC9627621; doi:10.3389/fmed.2022.922431)
Supplement: Supplementary file 1 [file Data_Sheet_1.pdf]

Supplementary Material

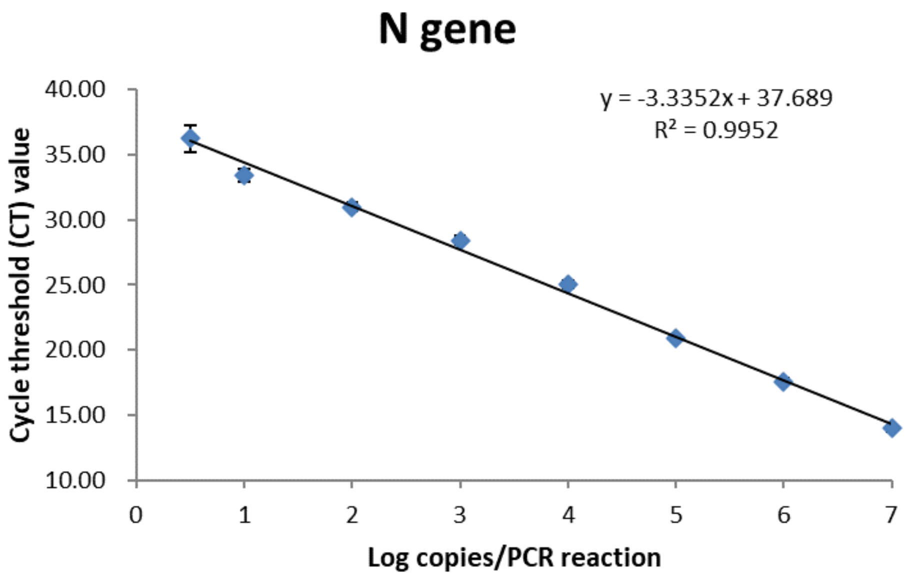

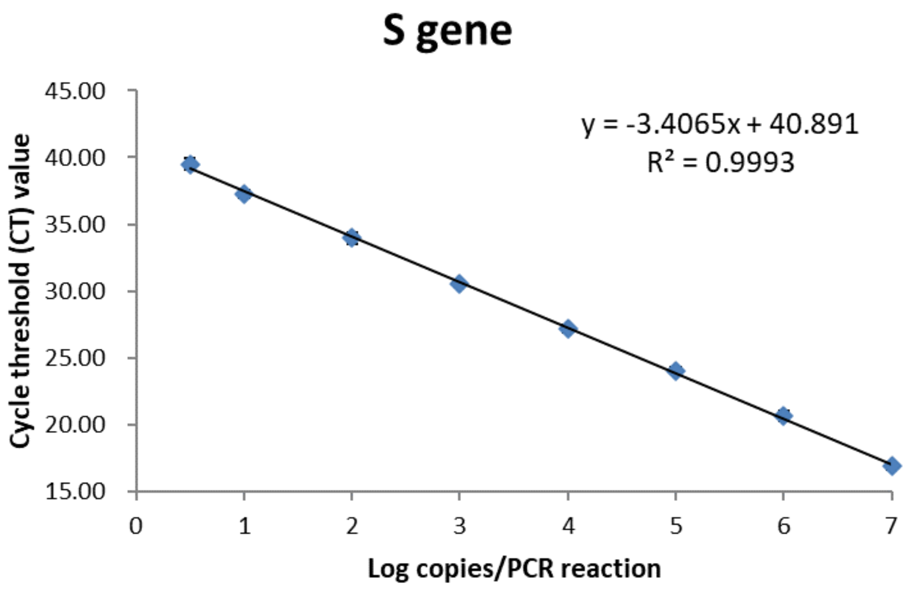

Supplementary Figure 1. Correlation Curves Between Cycle Thresholds and Viral Copy Number of SARS-CoV-2 N and S Genes

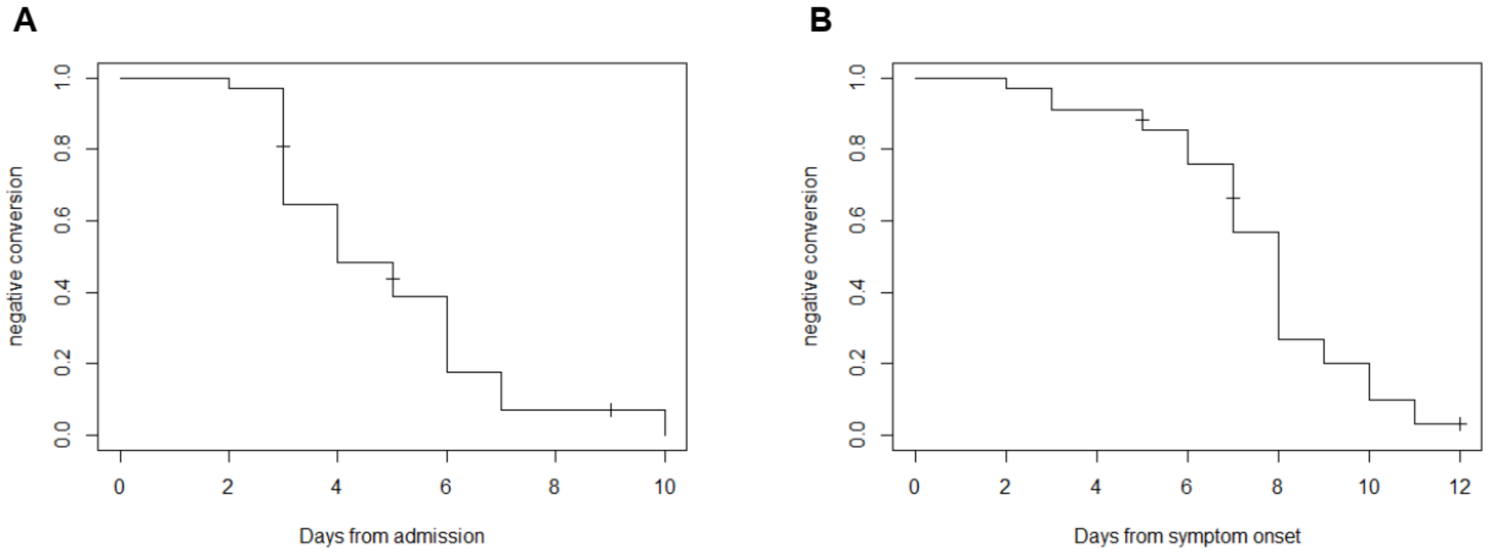

Supplementary Figure 2. Kaplan-Meier Plot for Negative Conversion of the Rapid Antigen Test. *A*. Kaplan-Meier curve for negative rapid antigen test resulting from admission at community treatment center. *B*. Kaplan-Meier curve for negative rapid antigen test resulting from symptom onset.

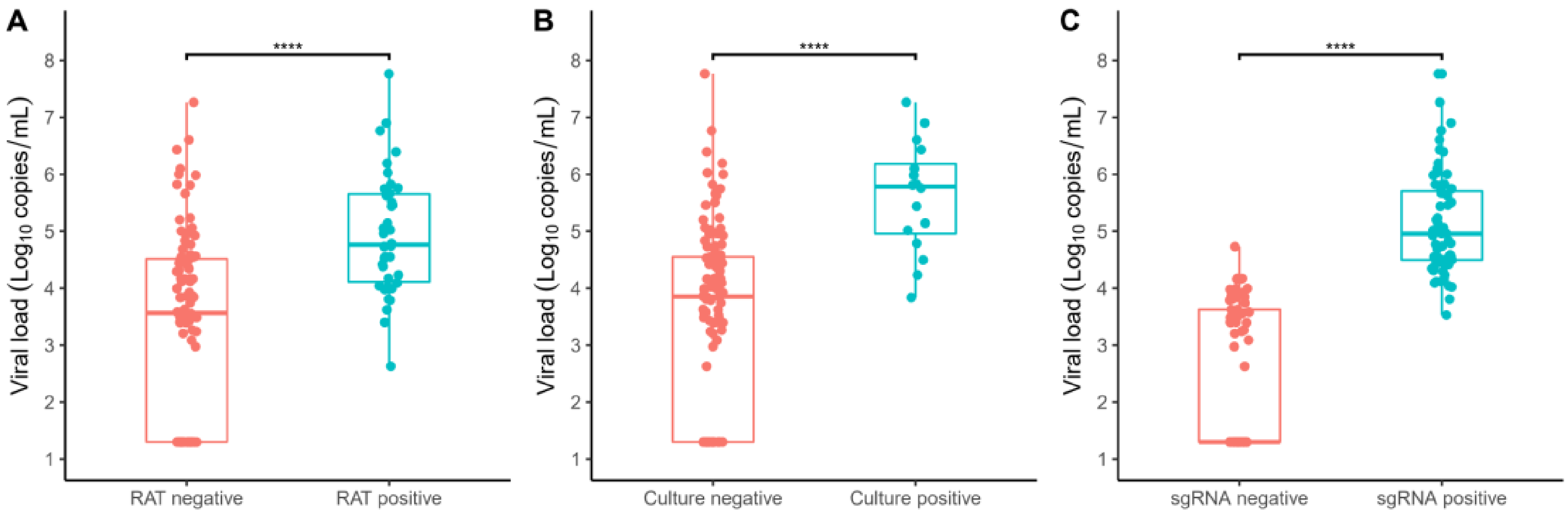

Supplementary Figure 3. Viral Loads According to Positivity of RAT (A), Culture (B), and Subgenomic RNA (C)

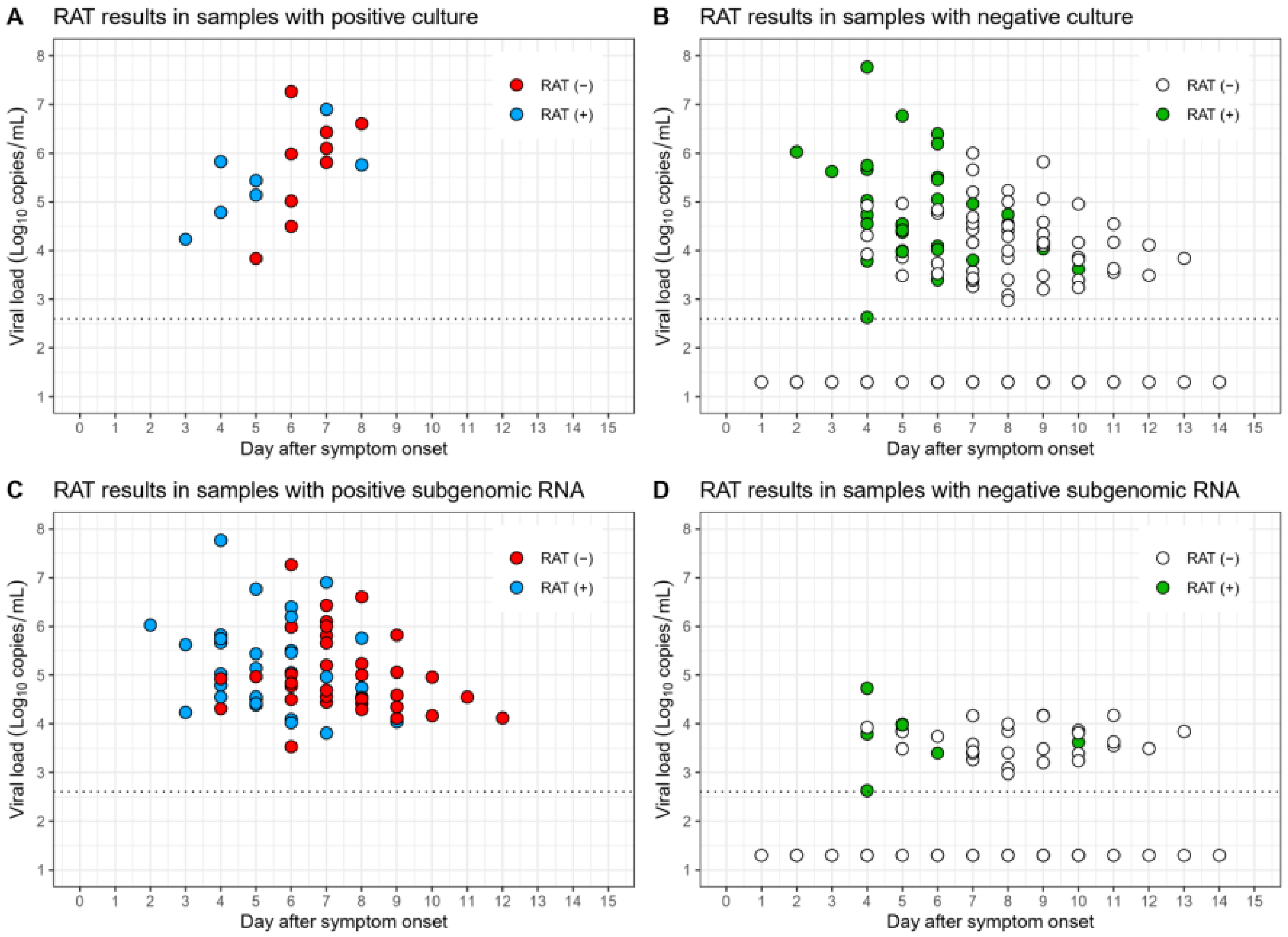

Supplementary Figure 4. Decomposed Scatter Plots According to Positivity of the Reference Test. *A*. Culture as the reference test. *B*. Subgenomic RNA as the reference test.

Hospital day

Patient	Age/Sex	1	2	3	4	5	6	7	8	9	10
1	46/M			X	X	X	X	X	X	X	
2	25/M			X				X	X	X	
3	30/F			O	X	X	X				
4	21/F			X	X						
5	31/M				O	O	X	X	X	X	
6	29/F			X	X	X	X	X	X	X	
7	20/M			O				X			
8	28/M			O	O	O		X			
9	34/M			X	X	X	X				
10	28/M			O							
11	23/M			X		X		X			
12	19/F			X							
13	21/F				O	O	O	X	X	X	
14	33/M			O	O	O	O	O	O	O	
15	43/M				X	X	X				
16	42/F			O	X	X	X	X			
17	48/F			X	X	X	X	X	X		
18	37/F		X	X	X	X					
19	30/F			X	O	O	X	X			
20	29/M			O	O	O		O	O		
21	29/F			X	X		X	X			
22	29/M			O							
23	39/F			O	O						
24	27/M			X	X	X	X	X			
25	25/M			O		X		X			
26	49/M						X		X	X	
27	27/M				X	X					
28	22/M					X	X	X	X		
29	27/F			O	X	X	X	X	X	X	X
30	39/M		O	O	O	X	X	X	X	X	
31	48/M				O	O	O	X	X	X	X
32	28/F						X	X			
33	49/M			X	X						
34	27/M			X							

Days after symptom onset

Patient	Age/Sex	0	1	2	3	4	5	6	7	8	9	10	11	12	13	14
1	46/M							X	X	X	X	X	X	X		
2	25/M						X				X	X	X			
3	30/F							O	X	X	X					
4	21/F					X	X									
5	31/M					O	O	X	X	X	X					
6	29/F			X	X	X	X	X	X	X						
7	20/M					O				X						
8	28/M							O	O	O		X				
9	34/M							X	X	X	X					
10	28/M					O										
11	23/M			X		X		X								
12	19/F						X									
13	21/F					O	O	O	X	X	X					
14	33/M				O	O	O	O	O	O	O					
15	43/M							X	X	X						
16	42/F					O	X	X	X	X						
17	48/F						X	X	X	X	X	X				
18	37/F		X	X	X	X										
19	30/F					X	O	O	X	X						
20	29/M						O	O	O		O	O				
21	29/F											X	X		X	X
22	29/M					O										
23	39/F					O	O									
24	27/M										X	X	X	X	X	
25	25/M			O		X		X								
26	49/M									X		X	X			
27	27/M							X	X							
28	22/M								X	X	X	X				
29	27/F						O	X	X	X	X	X	X	X		
30	39/M				O	O	O	X	X	X	X	X				
31	48/M					O	O	O	X	X	X	X				
32	28/F								X	X						
33	49/M									X	X					
34	27/M				X				X							

negative virus culture

positive virus culture

Supplementary Figure 5. Timeline of the test results of rapid-antigen test and viral culture. *A.* According to hospital day *B.* According to days after symptom onset. Test results of the rapid-antigen test were shown as “O” (positive) or “X” (negative).

Supplementary Table 1. Primers and Probes for Real-time RT-PCR Assay for Detection of N and S Genes of SARS-CoV-2

Target (Accession #)	Name	Location	Sequence (5'-3')	Modification
N gene (NC_045512)	NF	29356	AACATTCCCACCAACAGAGC	
	NR	29529	GCCTGAGTTGAGTCAGCACT	
	NP	29462	GCTGATGAAACTCAAGCCTTACCGCA	5'Cy5, 3'BHQ2
S gene (NC_045512)	SF	21624	GAACTCAATTACCCCCTGCAT	
	SR	21787	ACCATTGGTCCCAGAGACAT	
	SP	21657	TCACACGTGGTGTTTATTACCCTGACA	5'FAM, 3'BHQ1
Internal control (NC_000007.14)	BAF	1670	ACTAACACTGGCTCGTGTGA	
	BAR	1774	CTTGGGATGGGGAGTCTGTT	
	BAP	1700	AGGCTGGTGTAAAGCGGCCTTGG	5'HEX, 3'BHQ1

Supplementary Table 2. Primers and Probes for Detection of N and S Gene Subgenomic RNAs

Target*	Name	Location	Sequence (5’-3’)
5’ leader	SG-F	15	CCTTCCCAGGTAACAAACCA
N gene	SG-NR	28497	TTAATTGGAACGCCTTGTCC
S gene	SG-SR	21905	GGGACTGGGTCTTCGAATCT

(*Accession #: NC_045512.2)

Supplementary Table 3. Performance of subgenomic RNA and genomic RNA tests compared with viral culture.

	Sensitivity (95% CI)	Specificity (95% CI)	PPV (95% CI)	NPV (95% CI)
Subgenomic RNA vs. viral culture	94% (70–100)	57% (48–66)	22% (13–34)	99% (92–100)
Genomic RNA vs. viral culture	100% (79–100)	30% (22–38)	16% (09–24)	100% (90–100)

CI, confidence interval; PPV, positive predictive value; NPV, negative predictive value.
